# Neurofeedback of slow cortical potentials: neural mechanisms and feasibility of a placebo-controlled design in healthy adults

**DOI:** 10.3389/fnhum.2014.00990

**Published:** 2014-12-11

**Authors:** Holger Gevensleben, Björn Albrecht, Henry Lütcke, Tibor Auer, Wan Ilma Dewiputri, Renate Schweizer, Gunther Moll, Hartmut Heinrich, Aribert Rothenberger

**Affiliations:** ^1^Child and Adolescent Psychiatry, University Medical Center (UMG)Göttingen, Germany; ^2^Biomedizinische NMR Forschungs GmbH, MPI for Biophysical ChemistryGöttingen, Germany; ^3^Scientific IT Services, ETH ZürichZürich, Switzerland; ^4^3MRC Cognition and Brain Sciences Unit, University CambridgeCambridge, UK; ^5^Department of Neuroscience, School of Medical Sciences, Universiti Sains MalaysiaPulau Pinang, Malaysia; ^6^Department of Child and Adolescent Mental Health, University Hospital of ErlangenErlangen, Germany; ^7^kbo-Heckscher-KlinikumMunich, Germany

**Keywords:** neurofeedback, EEG-biofeedback, SCP training, fMRI, CNV, anterior cingulate cortex

## Abstract

To elucidate basic mechanisms underlying neurofeedback we investigated neural mechanisms of training of slow cortical potentials (SCPs) by considering EEG- and fMRI. Additionally, we analyzed the feasibility of a double-blind, placebo-controlled design in NF research based on regulation performance during treatment sessions and self-assessment of the participants. Twenty healthy adults participated in 16 sessions of SCPs training: 9 participants received regular SCP training, 11 participants received sham feedback. At three time points (pre, intermediate, post) fMRI and EEG/ERP-measurements were conducted during a continuous performance test (CPT). Performance-data during the sessions (regulation performance) in the treatment group and the placebo group were analyzed. Analysis of EEG-activity revealed in the SCP group a strong enhancement of the CNV (electrode Cz) at the intermediate assessment, followed by a decrease back to baseline at the post-treatment assessment. In contrast, in the placebo group a continuous but smaller increase of the CNV could be obtained from pre to post assessment. The increase of the CNV in the SCP group at intermediate testing was superior to the enhancement in the placebo group. The changes of the CNV were accompanied by a continuous improvement in the test performance of the CPT from pre to intermediate to post assessment comparable in both groups. The change of the CNV in the SCP group is interpreted as an indicator of neural plasticity and efficiency while an increase of the CNV in the placebo group might reflect learning and improved timing due to the frequent task repetition. In the fMRI analysis evidence was obtained for neuronal plasticity. After regular SCP neurofeedback activation in the posterior parietal cortex decreased from the pre- to the intermediate measurement and increased again in the post measurement, inversely following the U-shaped increase and decrease of the tCNV EEG amplitude in the SCP-trained group. Furthermore, we found a localized increase of activity in the anterior cingulate cortex (ACC). Analyses of the estimation of treatment assignment by the participants indicate feasibility of blinding. Participants could not assess treatment assignment confidently. Participants of the SCP-group improved regulation capability during treatment sessions (in contrast to the participants of the placebo-group), although regulation capability appeared to be instable, presumably due to diminished confidence in the training (SCP- or sham-training). Our results indicate that SCP training in healthy adults might lead to functional changes in neuronal circuits serving cognitive preparation even after a limited number of sessions.

## Introduction

Local cortical oscillations shape sensory, motor, and cognitive processes (Rothenberger, 2009). Such changes in neuroelectric activity are assumed to indicate the excitability of neuronal networks and have gained increasing interest in the investigation of mental functioning (e.g., executive functions, especially attention research, (Banaschewski and Brandeis, 2007; Calderone et al., 2014) as well as mental and emotional malfunctioning (Dennis, 2010; Henderson, 2010). In the search for neurophysiological conditions of (child- and adolescent) mental disorders different neuro-psychiatric disorders came into focus (Banaschewski and Brandeis, 2007). In first line, attention deficit/hyperactivity disorder (ADHD), epilepsy, and tic-/tourette disorder appeared to be associated with dysfunctions in the regulation of cortical excitation (Heinrich et al., 2007). Especially in children with ADHD, investigation of brain activity pattern do not only enrich the knowledge of neurophysiological concomitants of the disorder (for a review see Albrecht et al., under review) but built the theoretical background for neurofeedback as an innovative treatment tool, emerging from neurophysiological theory to clinical application (Pine, 2009). Recent randomized controlled trials document efficacy and clinical significance of neurofeedback in children with ADHD (Arns et al., 2014). Within a pool of different neurofeedback protocols applied in children with ADHD, SCP-training currently might be considered the best validated approach in this field (Mayer et al., 2013). However, mechanisms of action are neither on the neurobiological nor on the cognitive-behavioral level elucidated sufficiently (Gevensleben et al., 2014). A combination of EEG- and fMRI methodology within the scope of this double-blind, placebo-controlled study should give new insights in neurobiological and psychological mechanisms of SCP neurofeedback.

*Slow cortical potentials (SCPs)* are shifts in the cortical electrical activity lasting from several hundred milliseconds to several seconds. SCP might be externally triggered or self-induced. Their moderating impact on information processing has been demonstrated in numerous studies (Bauer and Nirnberger, 1981; Birbaumer et al., 1992; Schupp et al., 1994). Negative SCPs are assumed to reflect lowered thresholds for the excitation of underlying neuronal structures, leading to facilitation of processing e.g., during states of behavioral or cognitive preparation. Empirical evidence indicates e. g. accelerated reaction times during task performance (Lutzenberger et al., 1982; Rockstroh et al., 1982). Positive SCPs indicate reduction of cortical excitation of the underlying neural structures (e.g., during behavioral inhibition; Birbaumer et al., 1990), resulting e.g., in an attenuated startle reflex (Schupp et al., 1994).

The generation of a contingent negative variation (CNV), a characteristic negative SCP representing anticipatory attention, motivation, and motor preparation (Walter et al., 1964; Fan et al., 2007) relies on the activity of a thalamo-cortical-striatal circuit encompassing the prefrontal cortex (Rockstroh et al., 1993; Rosahl and Knight, 1995) primary and supplementary motor areas (Ioannides et al., 1994), posterior parietal cortex (Durstewitz, 2004) anterior cingulate cortex (ACC) and thalamic nuclei (Nagai et al., 2004). However, distinguishing early (initial, iCNV) and late (terminal, tCNV) components, different cortical and subcortical structures are assumed to be involved. This is indicated by differential source distributions for iCNV and tCNV in previous EEG studies. While the iCNV seems strongest at bilateral frontal electrodes, the tCNV appears to unfold maximum activity at the vertex (Birbaumer et al., 1990). Further evidence results from a previous trial of our laboratories. Using a continuous performance test (CPT) with a long inter-stimulus-interval (ISI) of 6 s, we found evidence for distinct cortical and subcortical brain regions associated with early and late components of the CNV (Lütcke et al., 2009). The late CNV mainly appeared to be associated with activations in the frontal cortex, dorsal ACC and thalamus and increased activity in midbrain dopaminergic nuclei (very likely corresponding to the substantia nigra). The initial CNV was localized mainly in motor and premotor cortical areas and the caudate nucleus.

Regulation of SCPs appears to be attenuated in children with ADHD, as indicated by a reduced CNV during CPT tasks (Banaschewski et al., 2003; Banaschewski and Brandeis, 2007). Furthermore, several controlled trials demonstrate that SCP training increases regulation of cortical excitability in terms of an enhanced post-treatment CNV and reduces ADHD symptomatology (Heinrich et al., 2004; Drechsler et al., 2007; Doehnert et al., 2008; Gevensleben et al., 2009, 2010; Mayer et al., 2013). Concerning children with ADHD, a more pronounced CNV seems to predict better outcome of a SCP training (Wangler et al., 2011).

Beyond neurobiological considerations, psychological (cognitive behavioral) operators or mechanisms of neurofeedback are hypothesized, but not empirical validated. Generation of SCP regulation capability (learning of neuro-regulation of SCPs) is assumed to rely on operant learning mechanisms, sharing pathways with skill motor acquisition (Strehl, this issue; Birbaumer et al., 2013). In how far effort, attributions, motivation or personality factors contribute to the outcome of SCP treatment is not sufficiently elucidated, although there is some evidence that there is an impact of such mental pattern (Gevensleben et al., 2014). It will take several trials to investigate the selective influence of distinct cognitive-behavioral (and emotional?) variables on the efficacy of different neurofeedback protocols. In the short run it seems important to get a rough idea of the impact of e. g. attributions (expectations, individual evaluations) on the course of neurofeedback training in order to distinguish valid from invalid strategies in the evaluation of treatment efficacy of NF. There is “pestering” request for double-blind, placebo-controlled trials, although the proof of feasibility in neurofeedback research is still weak, especially in children with ADHD. The NF-procedures (protocols and applications) used in those placebo-NF trials have been criticized for several reasons (inter alia poor treatment fidelity) and may account for the contradictory outcome (Arns et al., 2014; Gevensleben et al., 2014). Among other shortcomings of previous double-blind, placebo-controlled trials, none of the previous trials in children with ADHD could demonstrate validity of the treatment design in terms of “learning of neuro-regulation”. Acquisition of regulation capability during the treatment sessions is considered an indispensable prerequisite for a positive outcome of training. However, no previous placebo-trial could demonstrate learning. Contrariwise the latest placebo-trial in children with ADHD asserted that participant did not learn to regulate the targeted EEG parameters during treatment sessions (Vollebregt et al., 2014). Due to the fact that most participants of placebo-neurofeedback-trials consider the training a placebo treatment (even most of the participants of the “real treatment” group; Lansbergen et al., 2011; van Dongen-Boomsma et al., 2013) the lack of acquisition of regulation capability might result from impaired confidence in the treatment credibility during training.

In order to investigate neurobiological and psychological mechanisms of action of SCP training, we analyzed the impact of SCP training on the tCNV and conducted fMRI-whole brain analysis (parietal cortex ACC) in a CPT with long ISI. Using electroencephalography (EEG) and functional magnetic resonance imaging (fMRI) enabled us to investigate neural correlates of late anticipation (related to negative SCPs) at high temporal resolution (EEG) and at high spatial resolution (fMRI).

A second aim was to analyze the relation between the treatment evaluation (believe to get through a SCP- or placebo-training) and the acquisition of neuro-regulation capability during the training sessions.

## Materials and methods

### Subjects

Twenty healthy adults (age 18–29) participated in a SCP or sham NF-training, as well as in fMRI and EEG assessments. All experimental procedures conformed fully the institutional guidelines. The trial was approved by the local ethics committee of the University Medical Center Göttingen (UMG). Participants were informed about the purposes of the study and gave written informed consent. They were paid 85€ for the completion of the study. All participants were screened for mental/psychiatric disorders with the SKID-I screening questionnaire (Wittchen et al., 1997) supplemented by the assessment of symptoms of an attention deficit and hyperactivity disorder (Wender-Utah-Rating-Scale, short version; WURS-k; Retz-Junginger et al., 2002) and a general psychopathological profile (symptom-checklist, SCL-90-R; Leonard and Derogatis, 1994). General cognitive ability (GCA) was determined by the mean of four subtests of the WAIS-III (Wechsler, 1997; Table 1). There were no significant differences between the samples.

**Table 1:** **Demographic and clinical characteristics of the sample**.

Sample	SCP (*n* = 9) *M* (SD)	Placebo (*n* = 11) *M* (SD)
**Age** (years, month)	23.2 (2.91)	22.9 (2.98)
**Sex** (female/male)	7/2	
**GCA** (WAIS-III)	10 (2.56)	10.30 (1.44)
**WURS-k** (ADHD)	21.44 (9.15)	16.45 (4.30)
**SCL-90-R** (psychopathology)	0.15 (0.14)	0.29 (0.34)

*Description of the sample: GCA = mean of the four subtests vocabulary, bloc design, similarities, matrix reasoning of the WAIS-III; WURS-k: questionnaire which assesses symptoms of ADHD in childhood; SCL-90-R: GSI = global severity score (mean of all symptoms)*.

### Procedure

The study consisted of a SCP training and pre-, intermediate-, and post- training EEG and fMRI measurements. Subjects were, in a double-blind procedure, pseudo-randomly assigned to either real-SCP or sham-SCP training. Both trainings consisted of 16 training units of about 45 min each. Two units, divided by a short break, were conducted in each training session. The 8 training sessions were spread across 3 weeks, with generally two to three sessions per week depending on the schedule of the participants.

EEG- and fMRI-measurement were conducted before the first session (pre-test), after 4 sessions (intermediate-test), and after the final session (post-test). EEG measurements were performed in the EEG laboratory of the Department of Child and Adolescent Psychiatry, University Medical Center, Göttingen (UMG). FMRI measurements were performed at the Biomedizinische NMR Forschungs GmbH, Max Planck Institute for Biophysical Chemistry, Göttingen. The EEG and fMRI measurements at each time point were conducted within a week.

### Treatment (training)

The neurofeedback program SAM (“Self-regulation and Attention Management”) was used for both the SCP and the sham training. The SAM-system has been developed by our study group for scientific purposes and has been employed effectively in different previous NF studies (Heinrich et al., 2004; Drechsler et al., 2007; Gevensleben et al., 2009).

#### SCP training

Within the SAM training units, participants were asked to direct a ball on a computer screen upwards (negative SCP trials) or downwards (positive SCP trials) by generating negative or positive SCPs. All participants were instructed to get into an attentive (negative SCP trials) or relaxed state (positive SCP trials). Negative SCP and positive SCP trials were presented with equal probability in random order. One trial lasted for 8 s (baseline period: 2 s, feedback period: 6 s), inter-trial-interval was set to 5 ± 1 s. During the feedback phase, the mean SCP amplitude (moving time window: 1 s) was calculated at a rate of 10 Hz (10 times per second). Each SCP training unit presented approximately 120 trials and lasted 25–30 min. At least 1/3 transfer trials were conducted, where no feedback was provided. Transfer trials are thought to facilitate generalization (Heinrich et al., 2007).

Feedback was calculated from the Cz electrode, which is standard for SCP training (Heinrich et al., 2007; reference: mastoids, bandwidth: 0.01–30 Hz for SCP training, sampling rate: 250 Hz). Vertical eye movements, recorded from electrodes above and below the left eye, were corrected online using regression-based algorithms (Kotchoubey et al., 1997). Artifact thresholds were set to ± 100 μV in the EEG channel and ± 200 μV in the EOG channel. For segments containing artifacts exceeding this threshold no feedback was calculated. However, in individual cases thresholds were adapted (due to alternating signal quality, primarily at the beginning of the training) to enable contingent (less artifact-contaminated) feedback.

#### Sham training

In placebo training, the feedback data of participants of a previous study were used, providing an appropriate range of different feedback curves. These curves were weighted by coefficients to control the development of positive and negative SCPs in the course of the training such that participants should have the impression of the development of poor, average or good regulation skills over the course of the training. Three subjects (one third) of the placebo group were assigned to each of this “skill impression” group. Different approaches were taken to guarantee the blindness of the participants as well as of the trainers towards the training condition. Trainers did not see the online recorded EEG signal, but only the (real or simulated) feedback curve. Participants also saw the (real or simulated) feedback curve. For all participants (SCP and sham) the online recorded EOG signal was shown on the screen during the trials and the artifact detection was based in both training groups on the actual online EEG and EOG signals. This is considered to be an essential component to guarantee blindness of trainer and participant in a placebo-controlled study.

### Estimation of treatment assignment

On a five-point-scale (0 = “I strongly agree”; 1 = “I rather agree; 2 = “I don’t know; 3 = I rather disagree; 4 = “I strongly disagree”) participants rated their estimation of group assignment (“I’m involved in a regular neurofeedback training”) following each training sessions. The assessment controls for blinding and/or differences in the evaluation/estimation of the training. Furthermore the analysis of the guessed treatment assignment allowed investigating a potential relation between the estimation of the training and the development of regulation capability in the SCP group.

### Neuro-regulation assessment and analysis

During the training sessions subjects were instructed to generate shifts of cortical excitability (SCPs) towards positivity (reduced excitability) or negativity (enhanced excitability). Regulation indices were calculated as the difference between the EEG-activity during positivity trials vs. negativity trials, reflecting a measure of neuro-regulation capability. Due to the slow development of a SCP, only the last 4 s of the 6-s-feedback-interval of a trial were taken into account (Hinterberger et al., 2005). Analysis of regulation capability encompassed regular feedback as well as transfer trials combined.

The difference in the activity between positivity trials and negativity trials of one session in terms of a *regulation index* is considered as the regulation capability during a session.

The *session regulation index* describes the difference in the activity between positivity trials and negativity trials within one session. The *mean regulation index* represents the average of the in-session regulation indices of all 8 training sessions for each subject.

### Continuous performance task (CPT)

In the pre-, intermediate-, and post-training EEG and fMRI measurements, a cued version of a continuous performance task (CPT; van Leeuwen et al., 1998; Heinrich et al., 2004) with an extended stimulus onset asynchrony (SOA) of 6000 msec was applied. This duration closely corresponds to the standard duration of SCP trials during training sessions and conforms to the time resolution of the BOLD fMRI measurements (Lütcke et al., 2009).

For the CPT, subjects were presented with the letters O, X, or H. During EEG measurements black letters against a light gray background were shown in the center of a 17-inch CRT monitor with 800 × 600 points resolution against a light gray background at a viewing angle of 1.58 vertically and 1.08 horizontally. For fMRI measurement a dedicated setup was used (Schaefter and Kirchhoff, Hamburg, Germany) to project the stimuli on a screen within the MRI bore. Here black letters against a white background were presented. Two black vertical bars were continuously present above and below the stimulus location, to direct subjects’ attention to the center of the screen.

The letters were presented for 250 ms, with an inter stimulus interval of 5750 ms. The subjects were instructed that the letter O acted as an attentional signal (cue) and that they should press a response button as fast as possible with their right thumb or index finger if the following letter was an X (target) and to refrain from pressing the response button if the following letter was an H (distractor). To encourage fast responses, correct responses (button presses) had to occur within 1000 ms from stimulus onset. After the measurement subjects received visual feedback about the percentage of correct responses, as well as their average reaction time achieved.

A total of 80 stimuli were presented in one measurement (one block, total duration about 8 min.), the probability of an O-X pair (cued target) as well as the O-H pair (cued distractor) was 20% each (16 pairs/measurement). Additionally, there was a 10% chance of an uncued H (non-target) or X being shown. The test consisted of four blocks with a short break between each block.

### EEG recording and processing

Electrical activity of the brain was recorded with a BrainAmp amplifier (Brain Products, Munich, Germany) and sintered Ag/AgCl electrodes with Abralyt2000 electrolyte from 23 sites according to an extended 10–20 system (recording reference: FCz, ground electrode: CPz). Electrooculogram electrodes were placed above and below the right eye and at the outer canthi. Impedances of the electrodes were kept below 10 kOhm. Data was sampled at a rate of 500 Hz (bandwidth: 0.016–120 Hz).

Data were processed with Vision Analyzer software (Brain Products, Munich, Germany). Brain electrical activity was re-referenced to the average, and filtered offline with 0.05–30 Hz, 24 dB/oct Butterworth filters. Ocular artifacts were corrected by the methods described by Gratton et al. (1983). If the amplitude at any EEG electrode exceeded ±100 μV, a segment 150 ms before and 800 ms following was excluded from further analyzes. The Cue-related averages (−200–6500 ms) included at least 20 sweeps, and the tCNV was assessed as the mean amplitude 5000–6000 ms following cue onset at electrode Cz.

### fMRI imaging and data analysis

All MRI measurements were conducted at 3T (Siemens Tim Trio, Erlangen, Germany) using a 12-channel receive-only head coil. Individual structural T1-weighted MRI datasets were acquired using a 3D MP-RAGE sequence (1.3 × 1 × 1.3 mm^3^, interpolated to 1 × 1 × 1 mm^3^). fMRI was acquired with a single-shot, gradient-echo EPI sequence (TR = 2000 ms, TE = 36 ms, flip angle = 70°, 244 volumes per run) with a spatial resolution of 2 × 2 × 4 mm^3^ (matrix = 96 × 96, 192 mm FoV, 7/8 parial Fourier, bandwidth = 1336 Hz/pixel, echo spacing = 0.81 ms). 22 slices were acquired without gap in an interleaved fashion, positioned in the transvers-to-coronal plane, approximately parallel to the body of the corpus callosum and covering the whole cerebrum. To facilitate registration of fMRI data to the anatomical 3D image, one EPI volume with the same specifications as the functional series but with additional slices (36 slices) was acquired at the end of each fMRI session.

Evaluation of fMRI data was performed using tools from the FMRIB Software library (FSL).^1^ Scans were corrected for subject motion both in k-space (Siemens, Erlangen, Germany) as well as by image-based registration (Jenkinson et al., 2002). Nonbrain tissue was removed (Smith, 2002) and all volumes were intensity-normalized by the same factor and temporally high-pass filtered (Gaussian-weighted least-squares straight line fitting, with high-pass filter cutoff at 100 s). Data were smoothed using a Gaussian kernel of 5 mm FWHM. Boxcar models were convolved with a Gamma function to take into account temporal properties of the hemodynamic response (HR). Model fit was estimated by statistical time-series analysis in the framework of the general linear model (GLM) and with local autocorrelation correction (Woolrich et al., 2001).

First level regressors were describing the last 2 s before the presentation of the next letter in the cue (O-X, O-H) and the non-cue (H-O, H-X,) trials. Contrast of interests was set up as cue (32 trials) vs. non-cue trials (24 trials). This contrast emphasizes brain activation associated with anticipation, since subjects prepare for a possible reaction after the cue, but have no need for preparation in the non-cue trials. Contrast images were spatially normalized to the MNI152 template brain by means of their respective anatomical scan. Second-level fixed-effect analysis combined the 4 fMRI measurements within each session on the individual subject level. To summarize results across all subjects, mixed-effects group analysis was performed (Beckmann et al., 2003; Woolrich et al., 2004). Significant activations based on Z statistic (Gaussianized T/F) images were obtained by cluster thresholding determined by an initial threshold of *Z* > 2.3, and a corrected cluster significance threshold of *p* = 0.05 (Worsley et al., 1992). Group contrasts were set to compare brain activation changes from the pre- and intermediate-, the pre- and the post-, as well as the intermediate- and the post- measurements (TIME effect), between the groups of SCP- and placebo-trained subjects (GROUP effect) and the interaction between the two effects.

### EEG data analysis

The mean score of the *estimation of the assignment to the treatment* of the 8 training sessions was compared between both groups (*t*-test of independent samples) to control for blinding and/or differences in the evaluation/estimation of the training.

By comparing the difference of the *session regulation index* of session 1 with the *session regulation index* of session 8, intra-group development of regulation capability was analyzed for both groups (by paired *t*-tests). Mean regulation performance across all sessions between both groups was compared by independent *t*-test of the *mean regulation index* between both training groups.

The relation between the mean values of *estimation of the treatment assignment* and the mean regulation capability *(mean regulation index)* of the participants was analyzed by correlation analysis as well as the relation between the SCP in negativity trials in the single training sessions and the tCNV during the EEG-lab sessions (Pearson correlation coefficient).

CPT performance data (reaction time) in the EEG lab session and tCNV activation repeated measure ANOVAs (factor time: pre, int, post) was computed with group (SCP, sham) as between-subject factor.

Data analyses were performed using PASW Statistics (v.18).

## Results

### Estimation of treatment assignment

**Figure 1:**
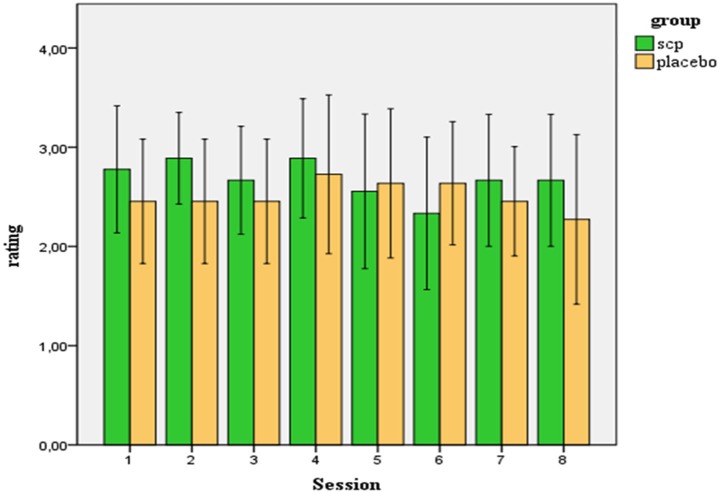
**Ratings (confidence intervals 95%) of the participants of how convinced they are to be involved in a true SCP training (in contrast to placebo training)**.

On a five-point-scale participants rated their estimation of group assignment. The average rating across all 20 participants was *M* = 2.61 (SD = 0.75). Considering the range of the scale from 0–4 (0 = “I strongly disagree to the estimation that I am involved in neurofeedback training”, 4 = “I strongly agree …”), the average estimation of the participants indicates ambivalence about the treatment condition. Ratings of both groups did not differ significantly, neither regarding the mean score across all sessions [SCP group: *M* = 2.73 (SD = 0.57), Placebo group: *M* = 2.52 (SD = 0.89); *t* = 0.62; *p* = 0.54)] nor concerning the rating after the last session, when the estimation of the treatment assignment should have been established by the participants [SCP group: *M*_S8_ = 2.67 (SD = 0.87); placebo group: *M*_S8_ = 2.27 (SD = 1.27); *t* = 0.79. *p* = 0.44]. In the end, across both groups, no change in the estimation of group affiliation across the sessions resulted [MANOVA: factor time: *F* = 0.66. *p* = 0.62; factor group: *F* = 0.24, *p* = 0.63; time × group: *F* = 0.87. *p* = 0.49]. Figure 1 illustrates the ratings of the SCP- and the placebo group across all sessions. Three subjects of the placebo group and no subject of the SCP group scored below “2” in the mean estimation of treatment assignment.

### SCP-regulation performance

For two subjects of the placebo group, session regulation data were lost due to hard disk problems. The analysis therefore encompassed SCP group = placebo group = 9 subjects. Due to organizational problems two subjects of the placebo group conducted only seven double-sessions. For these cases we chose a last-observation-carried-forward-approach (LOCF).

Find mean positivity and negativity for each session and session regulation indices in Table 2. Comparison of SCP amplitudes during positivity trials vs. negativity trials during the first session revealed no significant difference between both conditions, neither for the SCP group (*M*_pos1_ = 7.83; SD = 7.31; *M*_neg1_ = 3.42; SD = 5.98; *t* = 1.16. *p* = 0.28) nor for the placebo group (*M*_pos1_ = 10.16; SD = 8.78; *M*_neg1_ = 13.35; SD = 17.24; *t* = 0.77. *p* = 0.47). For the 8th session, a significant difference between positivity and negativity trials could be obtained in the SCP group (*M*_pos8_ = 6.84; SD = 7.31; *M*_neg8_ = −0.32; SD = 7.47; *t* = 2.73, *p* = 0.026) but not in the placebo group (*M*_pos8_ = 8.92; SD = 12.16; *M*_neg8_ = 7.10; SD = 8.08; *t* = 0.82, *p* = 0.44).

**Table 2:** **Regulation performance during SCP training sessions**.

Session	Regulation indices (μV) sessions 1–8						
	SCP group (*n* = 9)			Placebo group (*n* = 9)			Contrast
	Pos. (SD)	Neg. (SD)	Reg. (SD)	Pos. (SD)	Neg. (SD)	Reg. (SD)	Diff (p)
**1**	7.83 (7.31)	3.42 (5.98)	4.41 (11.37)	10.16 (8.78)	13.35 (17.24)	−3.19 (12.48)	7.60 (0.20)
**2**	3.29 (9.95)	0.69 (8.02)	2.60 (5.23)	7.45 (13.78)	6.40 (10.10)	1.05 (5.81)	1.55 (0.48)
**3**	2.15 (5.73)	1.83 (8.90)	0.32 (7.36)	5.58 (13.16)	8.68 (11.10)	−3.10 (5.95)	3.42 (0.16)
**4**	1.12 (8.31)	−3.51 (5.01)	4.64 (8.63)	3.06 (10.28)	5.38 (11.02)	−2.32 (5.05)	**6.96** (0.05)
**5**	4.88 (7.41)	0.76 (8.23)	4.12 (5.61)	10.39 (17.10)	13.58 (17.05)	−3.19 (11.35)	7.32 (0.10)
**6**	−1.53 (3.03)	−1.08 (6.00)	−0.45 (6.87)	6.97 (11.71)	7.52 (8.29)	−0.54 (6.07)	0.09 (0.98)
**7**	5.40 (5.63)	−1.22 (7.28)	6.62 (8.12)	1.96 (8.28)	4.10 (5.18)	−0.52 (6.82)	7.14 (0.06)
**8**	6.84 (8.84)	−0.32 (7.47)	7.16 (7.86)	6.35 (7.74)	5.79 (5.92)	1.82 (6.69)	5.34 (0.14)

Comparison of positivity trials, negativity trials, and session regulation indices (positivity trials − negativity trials) between both training groups for each session. Positive values of the regulation-indices indicate differences between negativity and positivity trials in the desired direction.

Comparing the mean regulation index (positivity—negativity trials across all sessions) reveals a significantly enhanced regulation capability in the SCP group (*M*_reg_ = 3.68, SD = 5.03) compared to the placebo group (*M*_reg_ = −1.25, SD = 3.85; *t* = 2.33, *p* = 0.03).

**Figure 2:**
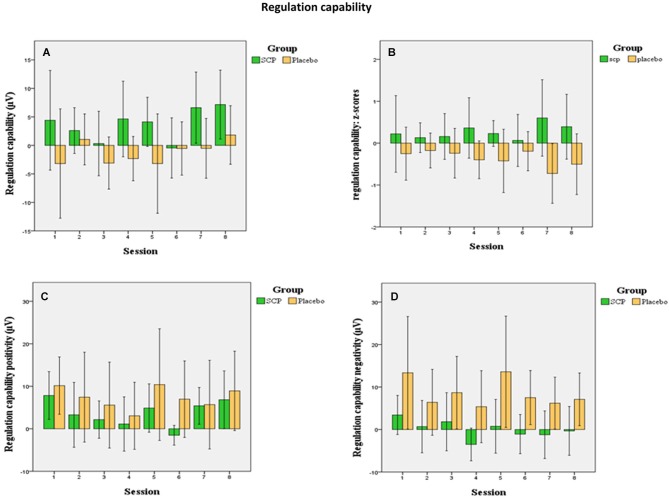
**Mean *session regulation indices* across all sessions (A) show an increase in the regulation capability in the SCP group (confidence intervals 95%)**. In the placebo group no significant development of regulation capability appears. This appears more evident in the illustration of the *z*-transformed (standardized deviation from the mean)* session regulation indices*. **(B)** Changes in regulation capability across all sessions for positivity trials **(C)** indicate no differences between the groups during positivity trials while only the SCP group develops the capability to generate negativity during negativity trials **(D)**.

Differences in regulation capability primarily result from enhanced activity during negativity trials in the SCP group (illustrated in Figure 2). There is no difference in mean activity during positivity trials across all sessions between both training groups (SCP group: *M*_pos_ = 3.75, SD = 3.15; placebo group: *M*_pos_ = 7.28, SD = 10.83), *t* = 0.94, *p* = 0.36), but significant more negativity during negativity trials in the SCP-group (SCP group: *M*_neg_ = 0.07, SD = 4.38; placebo group: *M*_neg_ = 8.53, SD = 9.56; *t* = 2.41, *p* = 0.03). Altogether, regulation capability evolves only in the SCP group and results from enhanced regulation toward negativity in negativity trials.

### Interrelation of regulation capability and estimation of treatment assignment

No significant relation between general regulation capability (*mean regulation index*) and mean estimation of group assignment (across all session) could be obtained in the SCP group (*r* = 0.04, *p* = 0.91). Table 3 presents Pearson correlation coefficients between the participants’ ratings of group assignment and the regulation performance for each session separately. No systematic relation between estimation of group assignment and regulation capability could be obtained, excluding two significant correlation coefficients for the sessions 4 and 5 concerning positivity regulation (session 4) and differentiation (session 5).

**Table 3:** **Correlation coefficients between rating of group assignment and regulation performance in the SCP group**.

*n* = 9	Rating session 1–8: correlation (p)							
Regulation	1	2	3	4	5	6	7	8
**Positivity**	−0.38 (0.32)	0.50 (0.17)	−0.41 (0.27)	**0.83 (0.01)**	0.58 (0.10)	0.40 (0.28)	0.04 (0.92)	0.27 (0.49)
**Negativity**	0.46 (0.21)	0.58 (0.10)	−0.18 (0.64)	0.24 (0.53)	0.02 (0.95)	0.27 (0.48)	−0.27 (0.48)	0.51 (0.16)
**Differentiation**	−0.48 (0.19)	0.05 (0.89)	−0.10 (0.80)	0.24 (0.53)	**0.74 (0.02)**	−0.06 (0.88)	0.27 (0.48)	−0.19 (0.63)

Pearson correlation coefficients for the SCP group between regulation performance (positivity, negativity, and differentiation = positivity − negativity) and the subjective ratings of the participants guessing the group assignment (SCP vs. placebo condition).

### Neurophysiological test session: tCNV and performance

**Figure 3:**
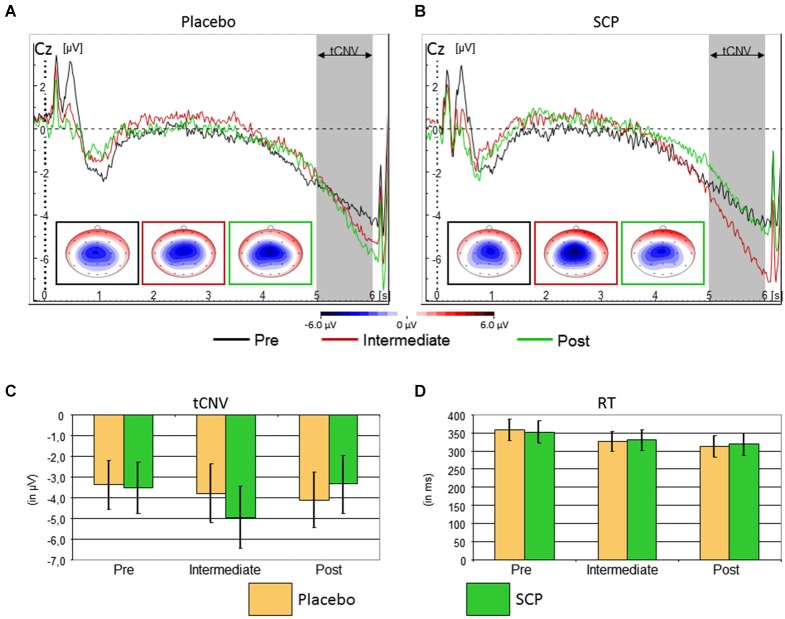
**Top:** Time course of the brain electrical activity related to cue processing at site Cz from the Placebo **(A)** and SCP **(B)** training groups at pre-training (black), intermediate (red) and post-training (green) assessment. The tCNV is assessed in the time window 5 to 6 s following cue onset and shows a central maximum. **Bottom:** Confidence intervals of tCNV and reaction-time (RT) with *p* = 0.05 for the comparison between Placebo and SCP training groups. The tCNV (with *p* = 0.05) displays a distinct time course throughout assessments: the Placebo training group exhibits as a tendency a steady increase in amplitude from pre-training to post-training assessment, whilst the SCP training group shows an inverted U-like shift with a significant and homogenous maximum at the Intermediate assessment **(C)**. Response speed became faster in later assessments in both training groups **(D)** CPT-performance in the neurophysiological test session was characterized by a generally high accuracy with on average less than 1.5% of omission and commission error rates in both SCP and Placebo training groups.

The event-related potential following cue stimuli showed the expected slow negative tCNV with a maximum at central leads that terminates with the onset of the next stimulus (see Figure 3). Exploratory analyses revealed that the maximum was located at electrode Cz where it was further evaluated. As illustrated in Figure 3, the tCNV mean amplitude shows distinct changes during the training course (Time: *F*_(2, 34)_ = 3.4, ɛ = 0.96, *p* = 0.05, part *η*^2^ = 0.17 and Training × Time: *F*_(2, 34)_ = 3.6, ɛ = 0.96, *p* = 0.04, part *η*^2^ = 0.18): whilst the Placebo group (Figure 3A) shows a tendency towards increased tCNV from Pre to Post assessment, the SCP group demonstrates a significant increase from Pre to Intermediate, and a significant decrease from Intermediate to Post, back to the Pre-training level (see Figures 3B,C).

Eight of nine subjects of the SCP group (but only the half of the placebo group) exhibited an enhancement of the CNV in the intermediate testing compared to initial measurement (pre-training testing), indicating that the intermediate CNV enhancement in the SCP group does not result from separate outliers.

Moderate to strong relations (correlation coefficients between 0.5 and 0.6) between the regulation performance (SCP during negativity trials) during single training session and the tCNV in the EEG-lab sessions developed, which however did not turn out to be significant due to the small sample size.

Response speed of correct responses showed a steady increase from pre to intermediate to the post assessment (Time: *F*_(2, 34)_ = 7.5, ɛ = 0.76, *p* < 0.01, part *η*^2^ = 0.31), which was similar for SCP and Placebo training (Training: *F*_(1, 17)_ < 1, *p* > 0.91, part *η*^2^ < 0.01, Training × Time: *F*_(2, 34)_ < 1, ɛ = 0.76, *p* = 0.76, part *η*^2^ < 0.01). There was further a marginal trend for an interaction “Block × Training” (*F*_(2, 34)_ = 2.2, ɛ = 0.78, *p* = 0.12, part *η*^2^ = 0.11), but *post hoc* tests revealed no clear differences in time-on-task effects across training groups. See Figure 3B for further details.

Response speed variability (RT-SD) was lower in the first compared to the later three blocks (Block: *F*_(3, 48)_ = 2.8, ɛ = 0.81, *p* = 0.06, part *η*^2^ = 0.15), but did not reveal any further effects (all *p* > 0.18).

### Functional MRI

**Figure 4:**
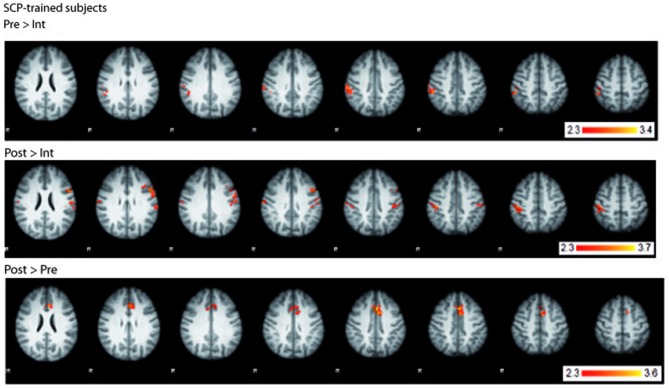
**BOLD-activation changes in the SCP-trained group across the training. Top:** BOLD-activation higher in the pre- compared to the intermediate measurement. **Middle:** BOLD-activation higher in the post- compared to the intermediate measurement. **Bottom:** BOLD-activation higher in the post- compared to the pre- measurement. *R* = right.

fMRI BOLD-activation of the last two seconds of the CPT anticipation phase was compared between SCP-trained and placebo-trained group for the three time points (pre, intermediate, post). All three analyses (pre vs. intermediate, intermediate vs. post, pre vs. post) showed no significant TIME × GROUP interaction in brain activation. However, computing within-group contrasts, three different BOLD-activation patterns became visible within the group of SCP-trained subjects (Figure 4) whereas no significant changes could be seen in the placebo group. In the comparison of the pre- and the intermediate measurement the SCP group showed lower BOLD-activation in the right parietal cortex (postcentral gyrus, peak coordinates: *x* = 50, *y* = −28, *z* = 56) and insular cortex (*x* = 32, *y* = 14, *z* = −4) at the Intermediate measurement. In the intermediate to post comparison, the BOLD-activation in the right and left parietal cortex (postcentral gyrus right: *x* = 48, *y* = −28, *z* = 48, postcentral gyrus left: *x* = −58, y = −16, *z* = 20) and insular cortex (*x* = 34, *y* = 16, *z* = 2) was again lower during the intermediate measurement. The pre-to-post comparison revealed a distinct increase in BOLD activation in the ACC (left ACC *x* = 0, *y* = 14, *z* = 40, right ACC *x* = 2, *y* = 20, *z* = 36) in the post measurement, which could also be detected in the overall pre-to-post comparison incorporating both, the SCP- and the placebo-group (main effect TIME).

## Discussion

### Neuronal plasticity: tCNV, fMRI, and performance

At the electrophysiological level we found a result somehow contrary to our primary expectations. The pre- to intermediate assessment in the SCP group revealed—according to our expectations—a strong increase of the tCNV during the CPT in the EEG-lab sessions. This increase in the SCP group significantly exceeded the increase of the tCNV in the placebo group. This enhancement of the parameter targeted by the SCP training (primarily related to negativity trials) was followed by a coequal tCNV decrease in the post-training assessment in the SCP group (back to baseline). Interestingly, this inverted U-like shift of the tCNV in the SCP group was accompanied by a continuing decrease in reaction time during CPT performance from pre- to intermediate assessment and from intermediate to post assessment. The continuing improvement in reaction time was comparable to the decrease of the reaction time in the placebo group. In contrast, in the placebo group the tCNV (as the assumed associated parameter of the performance on the neurophysiological level) showed a continuing increase in accordance with the decreasing reaction time. It appears that in the placebo group the increasing mobilization of neurophysiological resources (enhancement of the tCNV) is accompanied by coinciding improvement in the test performance (decreasing reaction time). Obviously the participants of the sham-group learned to optimize their CPT-performance, mobilizing more neurophysiological resources to continuously improve performance (learning/repetition effects). On the other hand, in the SCP group, the continuing improvement in reaction time is accompanied by an initial increase in the mobilization of neurophysiological resources in case of an enhanced tCNV in the intermediate testing, followed by reduction of neurophysiological effort accompanied by a further enhancement of the performance. Hence, it may be that subjects conducting SCP training require less neurophysiological resources to achieve a comparable performance in the long run (at post-training assessment).

This interpretation is in accordance with observations concerning e.g., professional musicians or elite athletes engaged in highly over-learned motor skill tasks. Long term practicing motor performance might lead to a more efficient generation of neural activity (reduced or more focused activity accompanying improvements in performance). In professional piano players motor areas were activated to a lesser degree during finger tapping tasks than in non-musicians (Jäncke et al., 2000; Krings et al., 2000). The same was true for Neymar (one of the most esteemed soccer players today), recruiting less resources in the motor-cortical regions controlling foot movement compared to less trained soccer players or athletes of other sporting disciplines, executing a simple foot movement task (Naito and Hirose, 2014).^2^ From the perspective of an athlete, this leaves a greater extent of motor cortical resources for accompanying or concurring motor tasks during the competition.

The decrease of the tCNV in the SCP group in the post-training assessment (compared to the intermediate testing) therefore might reflect the lesser effort which is needed after NF training to fulfill the same task with comparable adequacy. For the same task lesser neurons need to be activated (Krings et al., 2000). Hence, the same way that long term motor skill training induces plastic change in central motor systems, SCP training in healthy humans might result in reorganization of the cortical resource management, presumably leaving more resources for additional challenges.

Generally, the tCNV is considered to be associated with the negative SCPs which have to be generated during the SCP-sessions (Heinrich et al., 2004). We found some further support for this notion. The tCNV seemed to appear in relation to the development of the regulation performance during the training as indicated by moderate to strong (although non-significant) correlations between SCPs during negativity trials within the training sessions and the tCNV during EEG-lab sessions.

The development of the tCNV in the time course of this trial reveals results from previous trials studying the effects of SCP training in children with ADHD in a different light. The usual finding of SCP trials with children with ADHD is an enhanced (or less reduced) CNV after SCP training (Heinrich et al., 2004; Doehnert et al., 2008; Wangler et al., 2011). These are findings corresponding to the result of the intermediate assessment of our present trial with healthy adults. However, SCP regulation is impaired in children with ADHD (Banaschewski and Brandeis, 2007) so this might indicate, that even more sessions than usually practiced in research trials are necessary in children with ADHD to (firstly acquire an adequate regulation capability and) finally reach significant optimization on the neurophysiological level (on the other hand one might speculate that this optimization is seriously impaired in children with ADHD and might therefore not result even after a larger number of training sessions).

The additional whole brain data of the complementary fMRI CPT measurements may provide additional indications for the interpretation of the EEG results. Even if no significant difference in the GROUP × TIME interaction of the BOLD-activation could be detected between the groups of SCP- and placebo-trained subjects, significant changes in brain activation can be seen in the SCP-group across the time course of the training (within group contrasts) but not in the placebo-group. In the posterior parietal cortex activation, peaking at the right postcentral sulcus, decreases from the pre- to the intermediate measurement and, in the posterior parietal cortex of both hemispheres, increases again in the post measurement. This pattern inversely follows the U-shaped increase and decrease of the tCNV EEG amplitude in the SCP-trained group. Since the posterior parietal cortex is a multisensory motor association area, involved in motor planning (Andersen and Buneo, 2002), this change in BOLD-activation could reflect an aspect of the initial acquisition (pre to intermediate) and the following optimization process (intermediate to post) of the more efficient use of the neurophysiological resources. However, this interpretation should be taken with precaution, because these results are not significant in the overall GROUP × TIME interaction. The pre to post measurement increase in ACC BOLD-activation in the SCP-trained group, which is also seen in the overall contrast (main effect TIME), adds another facet, probably being more related to the changes induced by feedback processing during completing a neurofeedback (or placebo-) training. The ACC, being part of the decision making process in the frontal cortex (Rushworth et al., 2012), is also known to be specifically involved in processing of feedback signals to select the response, which is followed by a reward (Amiez et al., 2012; Rushworth et al., 2012). However, this change in ACC-activity was found in both groups and could therefore also reflect learning-/repetition effects of the CPT.

Taken together, for the SCP trained group, these BOLD-activation changes in two different areas of the brain, although being on the level of indications, provide some incidence that a successful training could not only involve multiple brain areas, but also encompass changes in different brain networks at different levels of optimization.

### Blinding, estimation of treatment assignment, and regulation capability

There is controversy about the feasibility of placebo-controlled trials in NF research. Firstly, previous trials failed to keep up blinding throughout the training and blinding came into question in placebo controlled NF (Holtmann et al., 2014) just as in psychopharmaceutical research, where as well blinding often might fail (Margraf et al., 1991; Morin et al., 1995). However, at least single-/double-blind, placebo controlled trials have been conducted with promising results concerning the application of blinding (and placebo control) in NF (Berner et al., 2006; Schabus et al., 2014). Secondly, placebo-control may affect fidelity of the training, e.g., diminish the credibility of the training or the effort spent by the participant (Gevensleben et al., 2012).

The estimations of our participants concerning the guessing of the treatment assignment display successful blinding. The estimation does not differ between SCP- and sham-training. The mean rating close to the middle of the rating scale reflects indecisiveness of the participants with little variance in the estimation. No significant correlation between estimation of group assignment and regulation performance could be obtained, making a significant influence of the estimation of the participants (as expected) quite improbable. However, there was not much variance in the estimations, making it hard to obtain an assumed connectivity between estimation and regulation capability. In further trials we would prefer to manipulate the estimation of the treatment directly (e.g., via opposed instructions).

Anecdotally we would like to note, that at least one participant (of the placebo-group) reported after completion of the trial that he was quite sure, that he had practiced placebo-training. He delineated his strategy that he once in a while reversed his regulation strategies but could not observe any systematic change in his displayed feedback following his switch of strategy. Additionally this underlines possible problems inherent in placebo-control in NF-research, participants spending effort in elucidating treatment assignment rather than struggling for enhanced regulation capability.

As expected, regulation capability developed differently in the SCP- compared to the placebo-group. While there was no difference in the generation of positivity during positivity trials between groups, the SCP-group learned to enhance negativity during the negativity trials (in contrast to the placebo-group). However there resulted no linear increase in regulation capability. Regulation performance in the SCP-group appeared to be instable with no significant differences in the regulation indices between SCP- and placebo-group for most of the sessions. However, acquisition of SCP-regulation capability is difficult (Neumann and Birbaumer, 2003) and probably further impaired by affected self-confidence and/or confidence in treatment credibility due to the implementation of a placebo-condition in this trial.

### Limitations and conclusions

The generalization of our results is limited by different factors among which we consider the most important the small sample size, which hardly allows for parametric testing. We consider the results as very relevant but preliminary and like to underline the need for replication with stronger sample sizes. The design of the study is compromised by the many repetitions of the CPT, due to the separated assessment of the EEG and fMRI measurement. This makes the test susceptible for learning processes overwriting or influencing systematic but sensible effects of the training. A combined EEG-fMRI assessment would significantly reduce the test repetitions and allow to directly put the EEG- in relation to the fMRI activity.

Nevertheless we consider these results a further step in understanding mechanisms of change in NF training, indicating neuronal plasticity even after a short number of SCP sessions although learning of SCP regulation does not appear to be optimal, probably due to blinding and uncertainty about the training condition (SCP or placebo).

## Conflict of interest statement

The authors declare that the research was conducted in the absence of any commercial or financial relationships that could be construed as a potential conflict of interest.

## Footnotes

^1^www.fmrib.ox.ac.uk

^2^This provides empirical evidence for the general impression that professional soccer players often do not make much use of their mental resources (at least while executing their complex movement patterns).
